# Tissue-Derived Stem Cell Research 2017

**DOI:** 10.1155/2018/8203537

**Published:** 2018-04-29

**Authors:** Ming Li, Kequan Guo, Luca Vanella

**Affiliations:** ^1^Laboratory of Cardiovascular Disease, Novel, Non-invasive, and Nutritional Therapeutics, Graduate School of Medicine, Osaka University, Osaka 5650874, Japan; ^2^Department of Cardiac Surgery, Beijing Institute of Heart, Lung & Blood Vessel Disease, Beijing Anzhen Hospital, Capital Medical University, Beijing 100029, China; ^3^Department of Drug Science, Section of Biochemistry, University of Catania, 95125 Catania, Italy

Tissue-derived stem cells (TDSCs), proven to be defined in tissues of bone marrow and blood vessels, adipose tissue, and placental tissue, have been highly approved as a particularly attractive autologous cell source for the purpose of regenerative therapy in the past decade. Accordingly, we opened up a special issue focused on TDSCs in 2016 with a large yield in appealing findings and correlated mechanisms though the safety aspects of their clinical applicability need to be well investigated and assessed in further study. In the meanwhile, the high risk of malignant transformation emerged as a critical obstacle to safe clinical translation of embryonic stem cell- or induced pluripotent stem cell-based therapies despite their fascinating differentiation potentials. Therefore, the compromising but feasible strategy of current regenerative therapies aimed at clinical applications is mostly limited to TDSCs isolated from adult tissue including bone marrow, adipose tissues, and placental tissues, which in comparison with totipotent stem cells have more specialization and less differentiation potential. In view of novel strategy and improved technique in the recent studies of TDSCs, we pushed forward a serial special issue in 2017.

Eighteen selected papers of research and review were published in this special issue according to the topics. In terms of the source of TDSCs, thirteen groups reported their studies in mesenchymal stem cells (MSCs) derived from bone marrow, adipose, lung, endometrium, placenta, and gingiva; three groups in stem cells derived from midbrain, limbal epithelial cell, and urine; one group in umbilical cord blood; and one group in hepatic progenitor cells.

Though the previous studies have demonstrated the efficacy of bone marrow-derived MSCs in graft-versus-host disease, osteogenesis imperfecta, myocardial infarction, systemic lupus erythematosus, and diabetes in both preclinical and early-phase clinical studies, the difference and the elucidating mechanisms of immune phenotype and proliferative potential between the bone marrow- and other source-derived MSCs and the modified technique were discussed in eleven papers of this special issue. D. Cappetta et al. reported that lung-derived MSCs improved emphysema induced by elastase in mouse. F. Lizcano et al. discussed that IL-4 receptor expressed in both precursor and adipocytes differentiated from human adipose-derived MSCs. Neuropilin 1 mediates keratinocyte growth factor-regulated adipose-derived MSC differentiation was reported in S. Ceccarelli et al.'s article. A. C. Yorukoglu et al. reviewed MSC sheets in osteogenic tissue engineering. Ž. Večerić-Haler et al.'s review focused on MSC therapy in acute kidney injury induced by cisplatin. D. Aboalola and V. K. M. Han discussed the effect of insulin-like growth factor-1 and 2 on myogenic differentiations from human MSCs. E. Lara et al. reported that PGE2 can modify the transcription profile of cattle endometrial MSCs. Y. Wanner et al. discussed that jaw platelet lysate was a better condition for culture jaw periosteal cells. F. Zhang et al.'s article indicated that inflammatory and anti-inflammatory synchronization affects stem cell regeneration from gingival stem/progenitor cells. D. Ye et al.'s article reported that transplanted MSCs may improve liver damage. N. Yusop et al. compared the characters of MSCs derived from rat bone marrow and the endosteal niche. M.-S. Chen et al. reported that IL-1 beta promotes MSC migration. M. Miyamoto et al. discussed that BMSC proliferates well in the recombinant peptide sponge.

Besides the studies of tissue-derived MSCs, interesting results were reported of other source-derived stem cells in four papers published in this special issue. T. Johansen et al. analysed calcium transients in the proliferation and differentiation of stem cell derived from human midbrain. Limbal epithelial cell culture condition was identified in E. K. Kim et al.'s article. X. Ji et al.'s review summarized the research of urine-derived stem cell. F. Li et al. discussed that Y-box protein can regulate the expression of collagen I in hepatic progenitor cells. A. Correa et al. reported that CD133-positive cells derived from human umbilical cord blood improved myocardial infarction.

In conclusion, it is entirely possible that TDSCs come to be the feasible and novel source into clinical regenerative trials in the near future encouraged by the fascinating observations from the original research articles as well as the review articles collected in this special issue. However, there still remain fundamental uncertainties including elucidation to mechanisms, safety aspects, and reproducibility regarding the variety of origin of stem cells.

